# Population Genetics and the Effects of a Severe Bottleneck in an *Ex Situ* Population of Critically Endangered Hawaiian Tree Snails

**DOI:** 10.1371/journal.pone.0114377

**Published:** 2014-12-03

**Authors:** Melissa R. Price, Michael G. Hadfield

**Affiliations:** 1 Pacific Biosciences Research Center, University of Hawai‘i at Mānoa, Honolulu, Hawai‘i, United States of America; 2 Kewalo Marine Laboratories, Honolulu, Hawai‘i, United States of America; Sichuan University, China

## Abstract

As wild populations decline, *ex situ* propagation provides a potential bank of genetic diversity and a hedge against extinction. These programs are unlikely to succeed if captive populations do not recover from the severe bottleneck imposed when they are founded with a limited number of individuals from remnant populations. In small captive populations allelic richness may be lost due to genetic drift, leading to a decline in fitness. Wild populations of the Hawaiian tree snail *Achatinella lila*, a hermaphroditic snail with a long life history, have declined precipitously due to introduced predators and other human impacts. A captive population initially thrived after its founding with seven snails, exceeding 600 captive individuals in 2009, but drastically declined in the last five years. Measures of fitness were examined from 2,018 captive snails that died between 1998 and 2012, and compared with genotypic data for six microsatellite loci from a subset of these deceased snails (*N* = 335), as well as live captive snails (*N* = 198) and wild snails (*N* = 92). Surprisingly, the inbreeding coefficient (*F_is_*) declined over time in the captive population, and is now approaching values observed in the 2013 wild population, despite a significant decrease in allelic richness. However, adult annual survival and fecundity significantly declined in the second generation. These measures of fitness were positively correlated with heterozygosity. Snails with higher measures of heterozygosity had more offspring, and third generation offspring with higher measures of heterozygosity were more likely to reach maturity. These results highlight the importance of maintaining genetic diversity in captive populations, particularly those initiated with a small number of individuals from wild remnant populations. Genetic rescue may allow for an increase in genetic diversity in the captive population, as measures of heterozygosity and rarified allelic richness were higher in wild tree snails.

## Introduction

Many species may be rescued from extinction only by *ex situ* or other managed breeding programs if the threat of introduced predators and continued habitat degradation persists [1]. However, few individuals may remain with which to found a captive-breeding program, resulting in a severe bottleneck when captive populations are initiated. Our understanding of the response of invertebrates, particularly molluscs, to severe bottlenecks is limited, yet these foundational species provide essential ecosystem functions, the loss of which will likely only be appreciated when they disappear [2].

Following a severe bottleneck, small captive populations of species with a long-life history and low fecundity are particularly vulnerable to synergistic interactions between demography and genetics. Hermaphroditic molluscs with high fecundity and the ability to self-fertilize may purge deleterious alleles and recover from a severe bottleneck [5]. Conversely, low fecundity may impede recovery in long-lived, late-maturing species. Deleterious alleles are more likely to accumulate in small populations, leading to declines in measures of fitness, such as survival and fecundity [4]. These lowered birth rates lessen the chance that deleterious mutations will be purged from the population, feeding back into their fixation [3].

The genus *Achatinella* consists of at least 41 species of hermaphroditic tree snails with long life spans, late maturity, and single, live births [6], [7]. Populations of these snails were once dense and abundant from sea level to at least 1500 m elevation, but today only fragmented, small populations remain. Habitat loss, predation by introduced species [7], and over-harvesting by collectors [8] led to the extinction of more than 30 species of *Achatinella*, and resulted in the declaration of all remaining species in the genus as Endangered [9].

**Figure 1:**
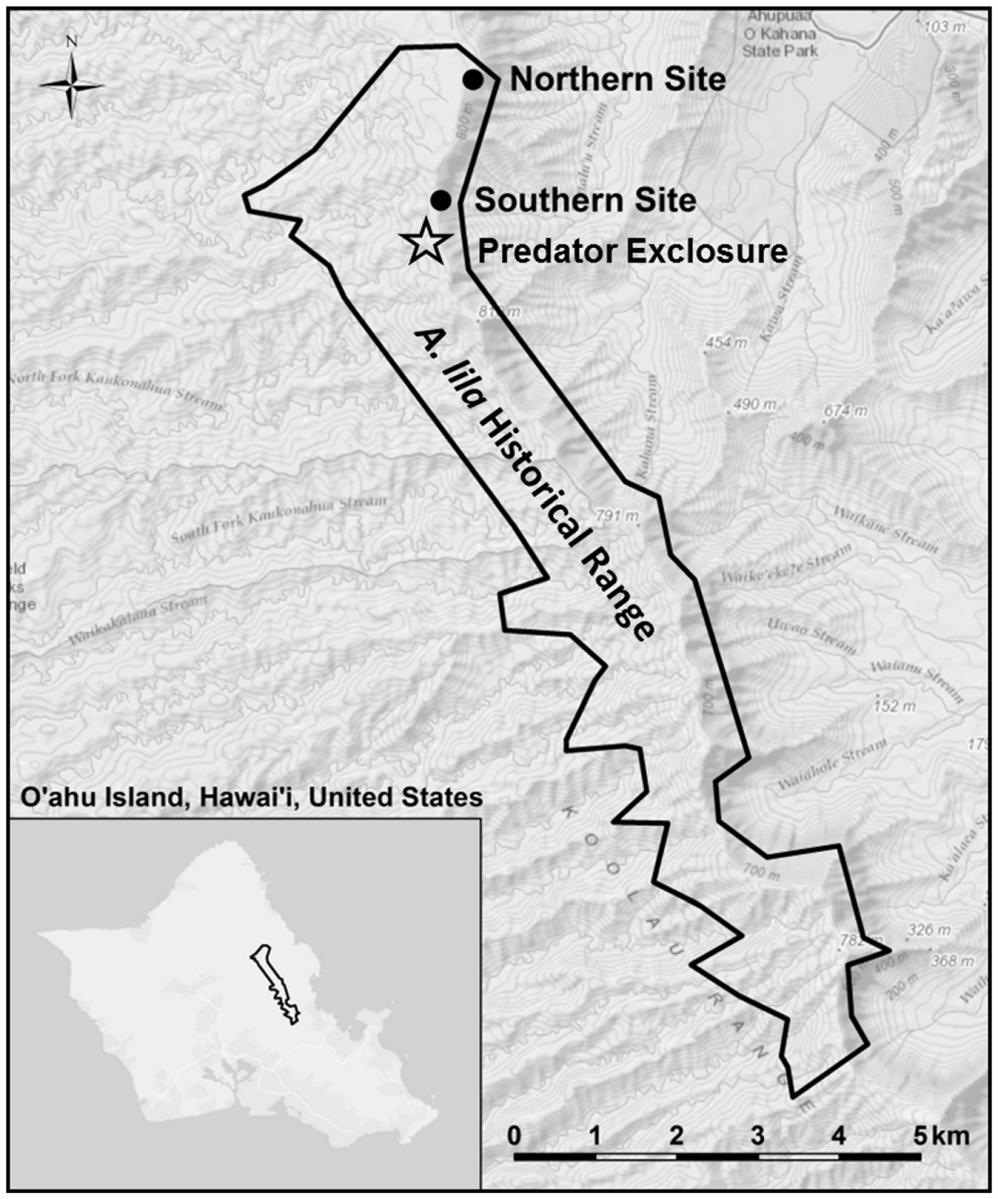
*Achatinella lila* historical range and two sampling locations. These two locations represent the only known populations that remain for this critically endangered species. The newly built predator exclosure will support *in situ* conservation efforts for wild snails endemic to this area.

One species of special concern is *A. lila*, with less than 200 known individuals remaining over less than five percent of its historic range (Fig 1). A captive population with seven snails was established in 1997 in the captive-rearing facility at the University of Hawai‘i at Mānoa as a hedge against extinction [6]. Following this constricted bottleneck, the population increased substantially from 1997 to 2009, reaching over 600 individuals, but is now in a five-year decline for unknown reasons. In this study we examined correlations between heterozygosity and several fitness measures including juvenile survival, survival to sexual maturity, and fecundity, and considered trends in the inbreeding coefficient (*F_is_*) over generations. We also compared genetic variation in wild *A. lila* with the captive population to provide long-term management recommendations.

## Materials and Methods

### Captive Breeding Facility and Demographic Records

The captive population of *A. lila* was founded in 1997 at the endemic Hawaiian tree snail captive-breeding facility at the University of Hawai‘i at Mānoa with seven adult snails collected and housed under USFWS permit PRT-826600. Sexual maturity is indicated by a lipped shell, or thickened edge of the shell aperture, at around 15 mm shell length in this species [10]. Once the lip is fully formed, shell growth ceases. Six of the founding snails were collected from a sampling location at the southern end of the species' range, and an additional snail came from the northern end of the range (Fig 1). The relatedness of the founders is unknown, but snails from the southern location were collected over a relatively small distance (less than 10 m).

Captive snails were reared in environmental chambers with controlled temperature and humidity designed to mimic wild conditions [6]. Hawaiian tree snails feed on an epiphytic fungal biofilm in the wild, so leafy branches of native plant species were placed in cages when they were cleaned biweekly, along with agar-cultured calcium-supplemented mold (*Cladosporium* sp.) originally isolated from a native snail-host plant [6]. At the time of cage cleaning, demographic information was collected including births, deaths, and total numbers of juveniles (shell length <9.5 mm), subadults (9.5 mm shell length to lipped shell), and adults. Individual snails were not labeled or tracked, but at death, each snail was individually preserved in 95% ethanol in a tube labeled with the species, shell length and width, source cage, and date of preservation (within 2 weeks of death).

Demographic data were collected from 2018 snails preserved between 1998 and 2013, 198 living snails in 2013, and from facility records. Using shell growth curves generated in captive *A. lila* [10], birth dates were estimated for both preserved and living captive snails. The number of births per year calculated using this method did not differ statistically from the number of births per year according to lab records (*t* = −0.49, *P* = 0.63). Birth dates, combined with shell length at the time of death, were used to calculate juvenile survival (snails surviving more than one year beyond their birth date) for the years 1998 through 2012. Based on published growth curves [10] and laboratory records, we added four years to the birth date to estimate the date of maturity. These data were used to estimate fecundity, or the number of offspring per adult, calculated by dividing the total number of offspring born in a particular year by the number of adults living during a particular year, for the years 1998 to 2012.

### Field Sites

*Achatinella lila* lives on native plants in forest dominated by O‘hia (*Metrosideros polymorpha*) in the northern Ko‘olau Mountain Range on the island of O‘ahu, Hawai‘i. Its known range currently extends two kilometers north to south along a constricted elevation of 700–850 m on the steep windward, moist slopes, where it co-occurs with sister species *A. sowerbyana, A. byronii,* and *A. bulimoides* [11]. Field sites are located at the southern and northern extent of the species' current range (Fig 1). The southern population, from which six of the snails founding the captive population were collected, has decreased drastically in the last decade. Only 12 snails were observed at the southern site in 2013, and previously known populations further south are believed to be extirpated. Snails have disappeared from the leeward side of the northern site, from which the seventh snail was collected, but remain on the windward slopes with a population size of approximately 100–200 snails.

### Sample Collection, DNA Extraction and Amplification

Only non-lethal, well-established, previously published methods were used for tissue collection [11], under permit TES-826600-12. Tissue samples were collected from all preserved snails that died between 1997 and 2001, and alternate years for preserved snails that died from 2002 to 2012, using sterile techniques (Table 1). Approximately 30–60 snails per year of death were selected based on the presence and quality of preserved tissue. For live wild and captive snails larger than 12 mm in shell length, samples were collected in late 2012 and early 2013 by cutting a very thin slice of tissue from the posterior tip of the foot, following a nonlethal sampling method [11], and stored in 100% ethanol for subsequent DNA extraction. DNA was extracted from tissue samples using a DNeasy Blood and Tissue Kit (Qiagen) according to the manufacturer's protocol, and eluted using two 200 µl washes of elution buffer (10 mM Tris-Cl, 0.5 mM EDTA). Wild and captive snails smaller than 12 mm were sampled by swabbing mucus from the snail's body using a sterile polyester-tipped swab, and storing the tip of the swab in a sterile, dry tube at −20°C until extraction. DNA was extracted from mucus using a QIAamp DNA Micro Kit (Qiagen) according to the manufacturer's protocol. Carrier RNA was added to the cell lysis buffer according to the manufacturer's protocol for very small amounts of DNA, and DNA was eluted in 50 µl of elution buffer.

**Table 1:** Distribution of *Achatinella lila* samples among collection years and source locations.

Source	*N*
**Captive breeding facility**	
Preserved snails	
1998–2001	7
2002	42
2004	40
2006	52
2008	37
2010	65
2012	92
Living snails 2013	198
**Wild snails**	
Southern site 2012	5
Northern site 2013	87

Each sample was genotyped at eight previously identified polymorphic microsatellite loci [12] using the recommended amplification protocols for each primer set, with the following adjustment. Bovine serum albumin was added to amplification reactions in increasing concentrations of 1 µM, 4 µM, and 10 µM for preserved specimens that failed to amplify, likely due to the presence of hemocyanin derivatives [13]. Genotyping reactions were performed by the Center for Genomic, Proteomic, and Bioinformatic Research (CGPBR) at the University of Hawai‘i at Mānoa. Amplification, genotyping and scoring were performed at least twice for each individual at all loci. Peakscanner version 1.0 (Applied Biosystems) was used to visualize and identify alleles.

### Statistical Analysis

Genotypic data were collected from 335 preserved captive, 198 live-sampled captive and 92 wild *A. lila* (Table 1). Scoring problems and null alleles were assessed using the software MICROCHECKER [14]. Null alleles and inbreeding may both result in data with excess homozygotes, the former due to large allele dropout, alleles identical in length but not by descent, or when alleles fail to amplify due to mutations in the flanking regions, and the latter due to an increased chance of both parents carrying the same allele [15]. To adjust for this potential discrepancy, null allele frequencies also were evaluated using the program INEst [16], with 10,000 iterations. This program compares several loci at the same time to simultaneously estimate null allele frequencies for individual loci and a mean inbreeding coefficient across loci [16]. By using more than one program to assess the presence of null alleles, false positives are minimized [15]. Based on the combined results generated by MICROCHECKER and INEst, two loci were dropped from subsequent analysis; one due to a high probability of null alleles, consistent with our observation that this locus inconsistently amplified across samples, and another with very low polymorphism. The six remaining loci amplified consistently across samples, were polymorphic, and had minimal null allele frequencies. Results from individual samples that failed to amplify at three or more loci were discarded prior to analysis.

The total captive population, including both deceased and living snails between 1997 and 2013, was divided into founder, F_1_, F_2_, and F_3_ generations for analysis. Generation time (T) was calculated as 5.59 years according to the equation M – 1 + (1/(1-v) [17], where M is the approximate maturation time (4 years), and v is the mean adult survival (0.614), or the number of adult snails surviving each year divided by the total number of adult snails alive during that year, averaged for the years 1997 to 2012. This resulted in F_1_ offspring born in years 1997 to 2002, F_2_ offspring born in years 2003 to 2008, and F_3_ offspring born in years 2009 to 2014. Snails brought into captivity in 1997, all of which died by the end of 2002, were referred to as the “founder” generation. Statistical tests were performed using the statistical package JMP (10.0). Fecundity was compared among generations using a Kruskall-Wallis nonparametric test followed by a nonparametric Wilcoxon test between each pair of generations. Changes in juvenile survival and survival to maturity over time were assessed using logistic regression analysis. Adult survival, or the proportion of adults surviving each year, was tested over time using linear regression analysis. Changes in survival to maturity among generations were tested using chi square analysis. Errors are reported as standard deviation.

Within generations born in captivity, we compared genetic measures for those that reached sexual maturity with those that did not reach sexual maturity. For the F_3_ generation, whose members were not old enough to have reached sexual maturity at the time of sampling, genetic measures were compared between deceased and living snails. Individual heterozygosity, calculated as the proportion of heterozygous loci, was arcsine transformed and compared among groups in the program JMP (10.0) using a *t*-test or an ANOVA, as appropriate, depending on the number of groups compared. Arlequin [18] was used to calculate genetic diversity statistics, including the inbreeding coefficient (*F_is_*), observed heterozygosity (*H_O_*), expected heterozygosity (*H_E_*), and genetic diversity.

To assess the relationship between individual fitness and heterozygosity, parentage was assigned for live-sampled captive offspring (<15 mm shell length) for all potential parents (adults ≥ 15 mm shell length) within the same cage, using the program Cervus version 3.03 [20]. Parentage assignments were then used to sum the number of living offspring produced per living adult, as a measure of individual adult fecundity. Relationships among fecundity, cage F_IS_, length, and arcsine-transformed individual heterozygosity (proportion of heterozygous loci) were tested with linear regression analysis using the statistical package JMP version 10.0.

Population differentiation (*F_ST_*) was assessed between the captive live population and the wild population with the software package Arlequin [18], using analysis of molecular variance (AMOVA) to evaluate the degree of population differentiation based on the relative number of repeats. Rarified allelic richness (*A_R_*) for a minimum sample size of *n* = 86 gene copies (43 diploid snails) was calculated using HP Rare, a program that implements rarefaction to control for sample size variation among groups [19]. Rarefied allelic richness was compared at six loci between wild snails and captive live snails, and between living captive snails and deceased captive snails with a Wilcoxon signed-rank test.

## Results

### Demographic Change

**Figure 2:**
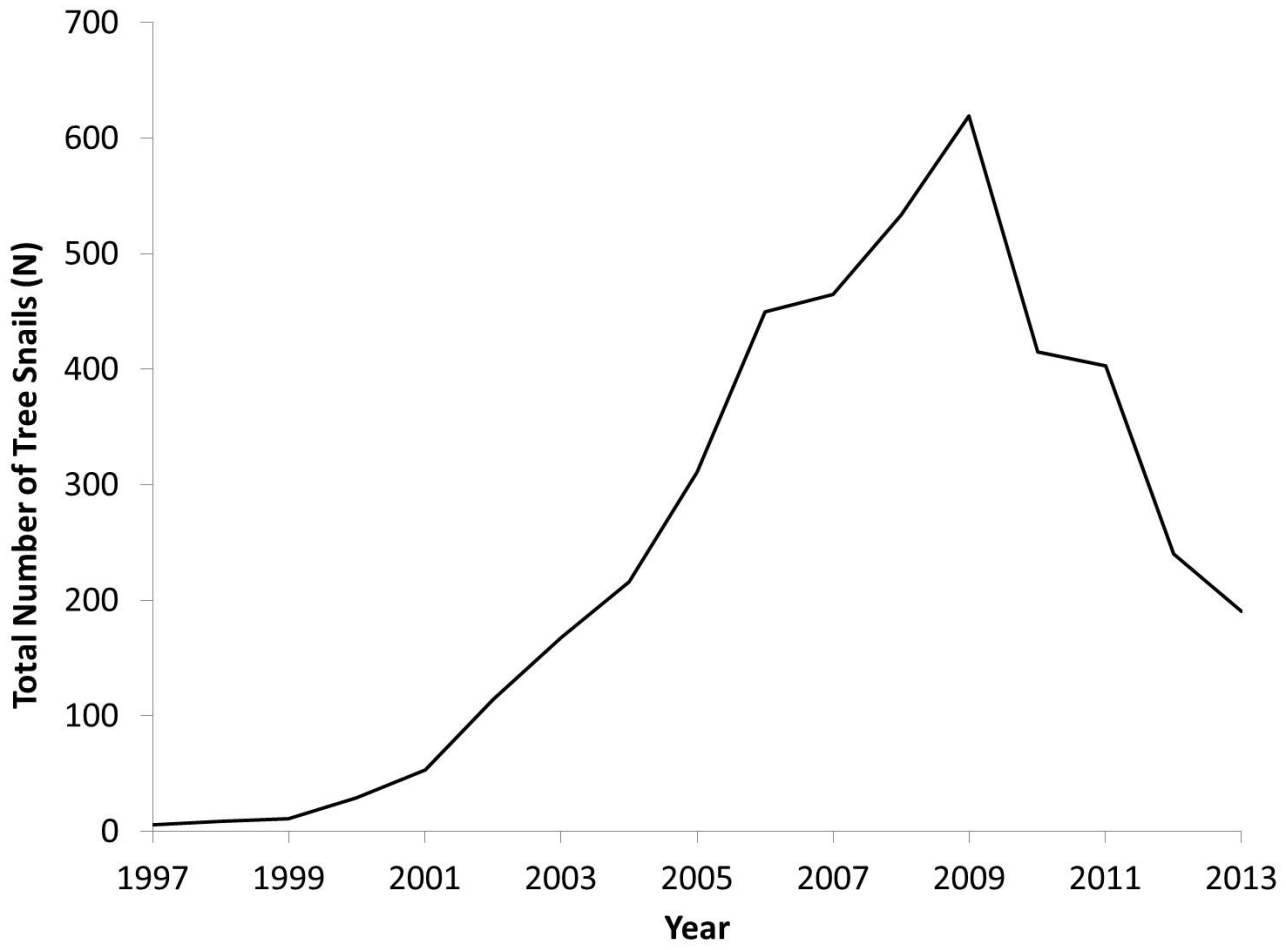
*Achatinella lila* captive population growth after its founding in 1997 with seven snails. The population peaked in 2009 with 668 snails and declined to less than 200 snails by 2013.

**Figure 3:**
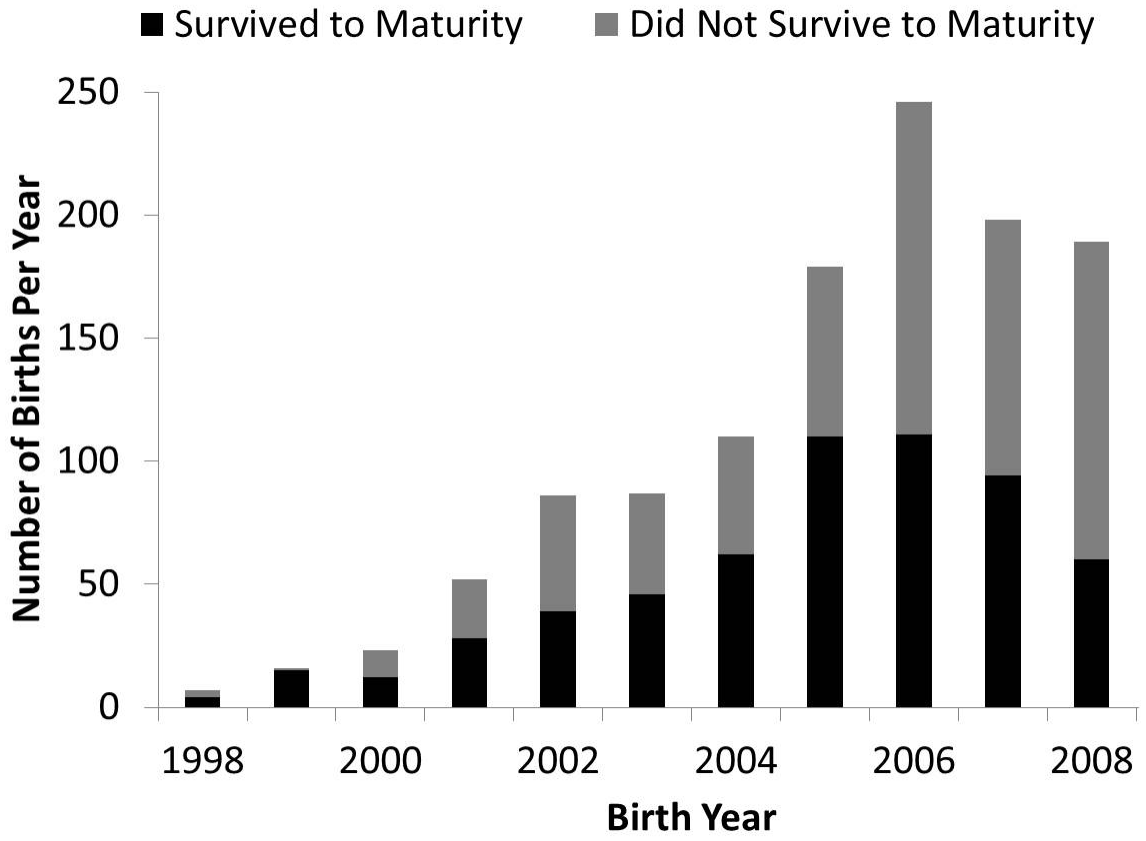
Proportion of snails born each year that survived to maturity for captive *Achatinella lila* from 1998 to 2008. Snails of this species reach maturity at approximately four years of age. The sum of snails that survived to maturity (black) and snails that did not survive to maturity (gray) indicates the total number of snails born in a given year. The number of births and the number of snails surviving to maturity peaked in 2006.

The captive population of *A. lila* grew substantially to a peak of 668 snails in 2009 (Fig 2). The seven founding snails died by 2002, limiting overlap between founders and sexually mature individuals from the F_1_ generation, since sexual maturity is not reached until about four years of age. A second bottleneck in the number of adults occurred in 2007, when the loss of 59 adults in a single year left only seven adults, again minimizing overlap between F_1_ and F_2_ adults. The total number of births and number of individuals surviving to maturity increased through 2006, then began a steady decline that continues to the present (Fig 3).

**Figure 4:**
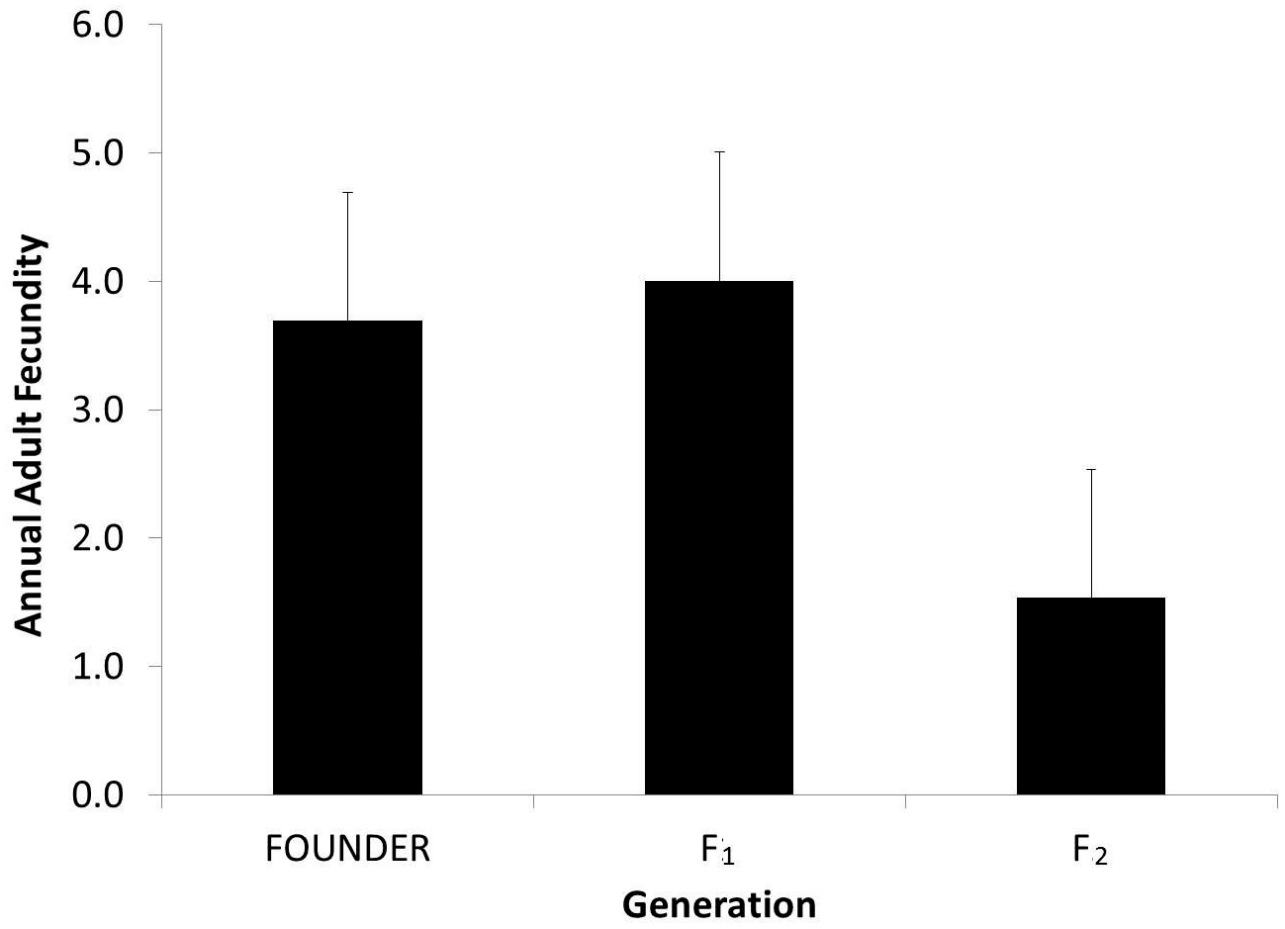
Fecundity significantly declined in the second generation of adults in the captive population of *Achatinella lila*, but did not differ between the founders of the captive population and the first generation of adults.

Fecundity did not differ between the founder (3.4±0.7) and F_1_ (4.0±0.6) adults (*Z* = 0.64, *P* = 0.52, but decreased significantly between the F_1_ and F_2_ (1.5±0.6) generations (*Z* = 2.47, *P* = 0.014; Fig 4). Parentage assignment analysis identified a range of 0–8 (1.96±1.8) total living offspring per adult for the cohort live-sampled in 2012 and 2013, which included offspring up to three years of age.

All measures of survival including survival to maturity (*X^2^_1_* = 177.4, *P* <0.0001), juvenile survival (*X^2^_1_* = 132.2, *P* <0.0001), and adult survivorship (*r^2^* = 0.57, *P* <0.01) significantly declined over time, but the magnitude of decline varied among generations. The proportion of snails surviving to maturity did not significantly differ between the F_1_ (0.53) and F_2_ (0.50) generations (*X^2^_1_* = 133.7, *P* <0.0001). Juvenile survival was above 60 percent for the F_1_ (67%) and F_2_ (65%) generations, but declined to 27 percent in the F_3_ generation (*X^2^_2_* = 167.2, *P* <0.0001). Adult survivorship was highest from 1998 to 2002 (0.84±0.10), declined somewhat from 2003 to 2008 (0.59±0.09), and was lowest from 2009 to 2013 (0.43±0.23; (*X^2^_2_* = 6.68, *P* <0.035).

### Genetic Measures

Approximately 17 percent (*N* = 335) of 2018 captive snails preserved between 1998 and 2012 were evaluated for genetic diversity measures, along with all living captive snails (*N* = 198), and wild snails from the southern (*N* = 5) and northern (*N* = 87) field sites (Table 1). Rarified allelic richness varied across loci and groups (Table 2). Allelic richness differed significantly between living captive snails and their deceased captive predecessors (*S* = 10.5, *P* = 0.031).

**Table 2:** Allelic information for eight microsatellite loci [14] examined in *Achatinella lila*.

Loci name (GenBank assession no.)	All captive		Live captive		Wild		Allelic potential	
	*N_A_*	Size range (bp)	*N_A_*	Size range (bp)	*N_A_*	Size range (bp)	*N_A_*	Size range (bp)
AS812 (EU119381)	10	227–245	7	227–243	7	227–243	10	227–243
AS32 (EU119382)	6	192–216	5	192–216	6	192–216	6	192–216
AS46 (EU119383)^a^	4	210–231	2	219–222	6	207–228	6	207–228
AS53 (EU119384)	13	176–276	5	216–252	17	196–264	17	196–264
AS61 (EU119385)	15	172–226	5	157–226	9	181–226	15	157–226
AS62 (EU119386)	9	208–240	8	216–240	6	216–236	9	216–240
AS82 (EU119387)	5	147–180	1	159	5	153–189	5	153–189
AS110 (EU119388)^b^	8	226–259	9	226–334	12	214–262	13	214–334

a Discarded due to minimal polymorphism in this species.

b Discarded due to a high probability of high-frequency null alleles.

The number of alleles (*N_A_*) and number of base pairs (bp) varied among loci. ‘All captive’ includes both preserved and living captive snails. ‘Allelic potential’ indicates the combined number of alleles for all living snails, both wild and captive.

**Figure 5:**
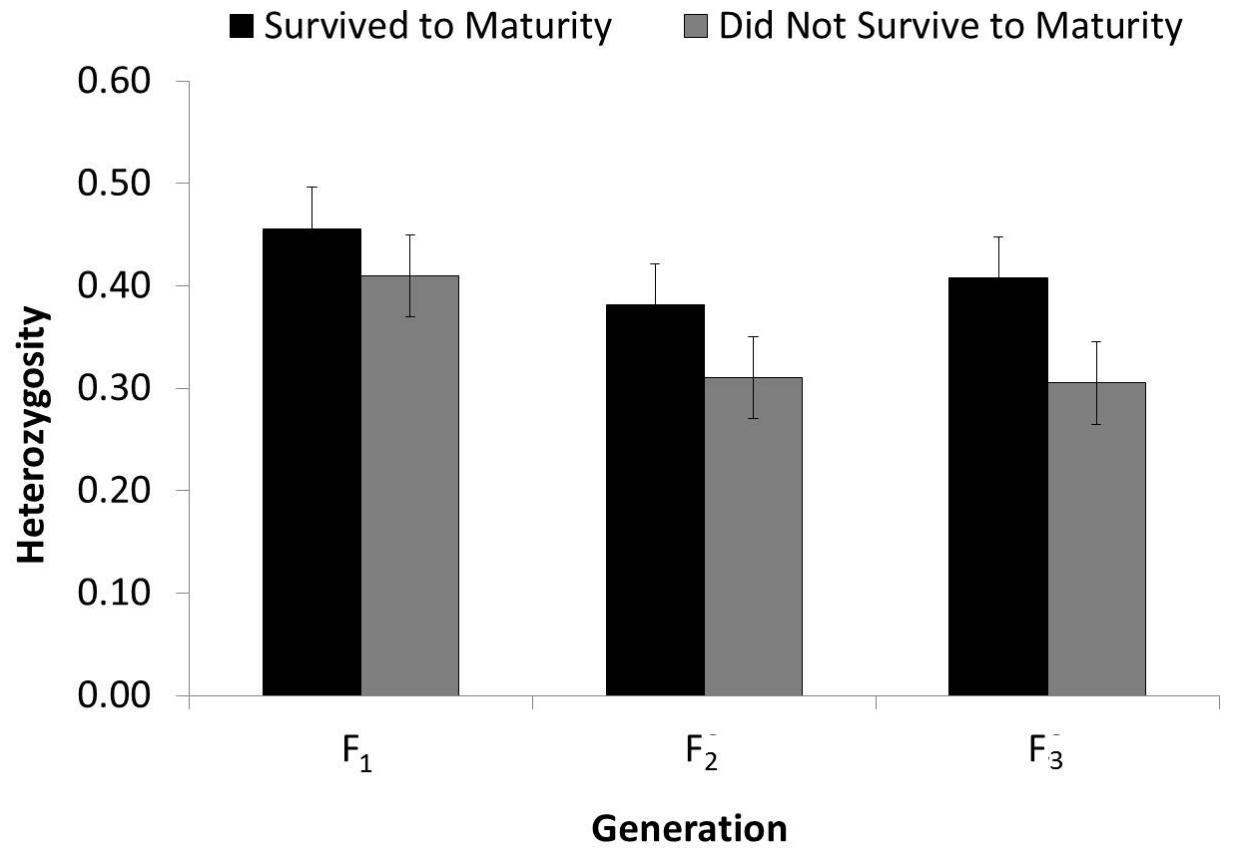
Heterozygosity was significantly higher in captive tree snails of *Achatinella lila* surviving to maturity in the third generation, but not the first or second.

The inbreeding coefficient (*F_is_*) decreased over time in snails reaching maturity, and snails that did not reach maturity had higher inbreeding values in each generation than those that did reach maturity (Table 3). Snails reaching maturity did not have significantly different heterozygosity than those that did not reach maturity in the F_1_ (*t* = 0.96, *P* = 0.34) or F_2_ (*t* = 1.03, *P* = 0.30) generations. In contrast, living snails from the F_3_ generation did have significantly higher heterozygosity than F_3_ deceased snails (*t* = 2.78, *P* <0.01; Fig 5). Snails live-sampled in 2013 with longer shell length did not have proportionally higher fecundity (*r^2^* <0.001, *P* = 0.99), but linear regression equations indicated that snails were likely to have approximately one less offspring for every 0.2 decrease in individual heterozygosity (*r^2^* = 0.15, *P* <0.001).

**Table 3:** Genetic measures for wild and captive *Achatinella lila*, including the number of individuals (*N*), Garza-Williamson bottleneck index (*M*), inbreeding coefficient (*F_is_*), observed heterozygosity (*H_O_*), expected heterozygosity (*H_E_*), gene diversity, and rarified allelic richness (*A_R_*), based on six microsatellite loci.

Generation and group	*N*	*M*	*F_IS_*	*H_O_* ± s.d.	*H_E_* ± s.d.	Gene diversity	*A_R_*
**Captive snails**							
Founders	6	0.37±0.15	0.395*	0.42±0.20	0.67±0.20	0.67±0.41	4.67±1.03
F_1_ (1997–2002)							
Reached maturity	43	0.50±0.20	0.367**	0.38±0.29	0.57±0.22	0.65±0.36	6.00±1.79
Died before maturity	60	0.58±0.18	0.380**	0.39±0.18	0.61±0.15	0.57±0.37	7.30±1.83
F_2_ (2003–2008)							
Reached maturity	154	0.42±0.08	0.388**	0.36±0.21	0.54±0.25	0.53±0.30	5.05±1.76
Died before maturity	89	0.57±0.16	0.456**	0.35±0.19	0.58±0.14	0.55±0.34	5.36±1.21
F_3_ (2009–2013)							
Live immature	125	0.53±0.21	0.258**	0.48±0.21	0.65±0.10	0.54±0.31	4.41±1.90
Deceased immature	56	0.56±0.13	0.366**	0.33±0.18	0.54±0.26	0.44±0.31	5.00±1.21
**Captive live 2013**							
Adult (> 4 yrs old)	73	0.64±0.23	0.313**	0.44±0.10	0.63±0.10	0.53±0.30	4.02±1.89
Subadult (1-4 yrs old)	80	0.65±0.23	0.268**	0.50±0.22	0.66±0.12	0.55±0.32	4.64±2.45
Juvenile (<1 yr old)	45	0.55±0.23	0.244**	0.47±0.20	0.65±0.10	0.54±0.31	4.16±1.74
**Wild 2013**	92	0.65±0.20	0.224**	0.53±0.13	0.69±0.15	0.68±0.38	7.52±4.49

^*^P <0.01 Significant departure from Hardy-Weinberg expectations at alpha = 0.01.

^**^P <0.001 Significant departure from Hardy-Weinberg expectations at alpha = 0.001.

Living and deceased snails were compared in the third generation, rather than mature and immature, as most of the snails in this generation did not have time to reach maturity prior to analysis.

The live-sampled captive snail population displayed significant genetic differentiation from the wild-sampled snail population (*F_ST_* = 0.23, *P* <0.001; Table 4). The live captive snails had fewer alleles than the wild snails at three of six loci, but allelic richness did not differ statistically between these groups (S = 5.50, *P* = 0.19). Wild snails (*F_IS(wild)_* = 0.224, *P* <0.001) had a similar inbreeding coefficient to live captive snails born in 2012 and 2013 (*F_IS(captive juvenile)_* = 0.244, *P* <0.001). Values for the Garza-Williamson bottleneck index were similar in wild snails (0.65±0.20) and live captive adults (0.64±0.23).

**Table 4:** Analysis of molecular variance (AMOVA) results comparing living wild and captive-bred snails of *Achatinella lila* sampled in 2013.

Source of variation	d.f.	Sum of squares	Variance components	Percentage of variation	*F_ST_*	*P*
Among populations	1	139.6	0.546 Va	23.3	0.23	<0.0001
Within populations	288	658.9	0.491 Vb	20.9		
Within individuals	290	379.0	1.310 Vc	55.8		

## Discussion

Declining measures of fitness and a loss of allelic diversity in a captive population of *A. lila* raise concerns about the probability of extinction due to synergistic interactions between demographics and genetics [21]. Species recovery appeared possible following the severe bottleneck at the founding of the captive population, given population growth prior to 2009. However, in recent years mortality sharply increased and less than a third of the population remains. Declining fecundity and survival to maturity continue to hinder population recovery.

### Heterozygosity-Fitness Correlations and Genetic Measures

Measures of fitness in the founding adults were comparable to wild populations of closely related tree snail species. The founders of the captive *A. lila* population demonstrated slightly lower fecundity, but higher juvenile survival than wild populations of sister species *A. mustelina* [22]. However, fecundity declined drastically after 2009, reaching a mean of approximately one offspring per adult per year. Survival to sexual maturity and adult annual survivorship declined significantly over time as well. Less than 25 percent of offspring born in the lab now reach maturity, and less than 50 percent of mature adults survive from year to year. In sum, all measures of fitness declined following 2009, despite decreases in measures of inbreeding (*F_is_*). Furthermore, measures of fitness were correlated with measures of heterozygosity. Snails with higher heterozygosity were more fecund and more likely to survive to sexual maturity.

Captive population allelic richness declined significantly over time, and is now lower, though not significantly so, in captive snails than in wild snails. A loss of allelic richness may interact with a number of other factors leading to the observed fitness declines in this captive population. Loss of genetic diversity is associated with an increased risk of mortality from multiple stressors [23]–[24], including infection [25]. Individuals with increased homozygosity may lack advantages conferred by heterozygosity (i.e., heterozygote advantage)[26], [27]. Alternatively, the limited number of breeders in a small population increases the chance for alleles to be identical by descent, leading to an increase in homozygosity for deleterious recessive alleles, such as those that might make individuals more susceptible to disease or environmental stress [28]. In laboratory chambers, where conditions were designed to minimize stress, fitness differences may not have been observed prior to 2009 if effects of deleterious alleles or a lack of heterozygote advantage were masked under benign conditions [28]. Consequently, if a stressor was introduced in 2009, it may have revealed deleterious alleles or a lack of heterozygote advantage in the captive population, resulting in differential survival [28]. If a population survives a stressful event, such as exposure to a pathogen, it may in fact lower the inbreeding coefficient [29], as was observed in this study following the 2009 decline.

Several potential stressors that may be responsible for the captive population decline have been examined. Density effects on fecundity were tested in *A. lila* and determined to be nonsignificant [30]. Transmission electron micrographs of tissues from recently deceased snails were examined, and determined to be without microsporidia (pers. obs.), which caused the extinction of another species of land snail kept by captive propagation [31]. This does not rule out the possibility of infection with a virus, bacterium, or other pathogen. Finally, temperature and humidity in the environmental chambers were tested and found to differ from the displayed settings. Thus, temperature and humidity may not have been optimal for fecundity, survival, and growth.

### Inbreeding and *F_IS_*

Significant inbreeding coefficients indicating departure from Hardy-Weinberg expectations were observed in all groups of *A. lila* tested. Bottleneck effects may not be observed until several generations after a bottleneck occurs, so these values are not likely due to the bottleneck at the initiation of the captive population. However, the documented history for achatinellid species indicates they have been in severe decline due to predation, habitat fragmentation, and over-collection in the wild for at least the last half century, if not longer [22]. Repeated surveys performed over the last 40 years [7] document the decline and disappearance of countless remnant populations and entire species, particularly since the introduction of *Euglandina rosea*, or the Rosy Wolf Snail, that has devastated Hawaii's endemic snails [32]. Molecular analysis of closely related *A. sowerbyana* also indicated the wild populations have experienced bottlenecks [33]. Given this history and the current distribution of the two remaining populations of *A. lila* relative to known historical distributions (Fig 1), we suggest the significant inbreeding coefficients observed in founding members of the captive population may be indicative of a long-term demographic reduction in the wild populations.

Self-fertilization could also inflate the inbreeding coefficient in this hermaphroditic species. Self-fertilization has only rarely been observed in *Achatinella* and its sister genus *Partulina*, with one known occurrence in *P. redfieldii* [6], and one case each suspected in *A. mustelina* and *A. fulgens* (pers. obs.), all from isolated individuals. Rates of self-fertilization likely vary among species in the subfamily Achatinellinae. Previous studies suggest a mixed-mating system [6], or even a lack of self-fertilization in some species [33]. Low density and population fragmentation in the wild may have resulted in an increase in the rate of self-fertilization in *A. lila*, or this species may have a higher intrinsic rate of self-fertilization than close relatives. Unfortunately, populations where some self-fertilization occurs may be at an additional disadvantage, building up mildly deleterious alleles through self-fertilization, and expressing them in outcrossing individuals [21].

Since the only two remaining wild populations of *A. lila* both show significant departure from Hardy-Weinberg expectations, as tested with the *F_IS_* statistic, there is no population available to provide a baseline for comparison. Therefore, we cannot say with certainty whether cumulative inbreeding, self-fertilization, or some other explanation is responsible for the significant *F_IS_* values observed in this study, particularly the high inbreeding coefficient observed in the founding snails. The presence of null alleles, or alleles that don’t amplify due to mutations in the flanking primer region but are nonetheless present, can also artificially raise the inbreeding coefficient. Null alleles can be statistically detected when amplification rates are lower than expected for a particular locus. Prior to analysis, we discarded one locus due to a high probability of null alleles, as well as a second locus due to minimal polymorphism, and have high confidence that the presence of null alleles is minimal in the remaining six loci.

### Management Implications and Future Work

Three strategies have been suggested to retain fitness and genetic diversity in captive populations: optimize heterozygosity through molecular tracking and managed breeding; minimize inbreeding through tracking individuals and their offspring; or create inbred pairings to purge deleterious alleles [34]. Our results suggest the latter is not an effective strategy, in agreement with previous work [34]. Outbreeding with snails from the wild population would likely increase heterozygosity, and may increase fecundity and reduce adult mortality [34].

Garza-Williamson bottleneck-index values (*M*) were nearly identical in the 2013 wild (0.65±0.20) and live (0.64±0.23) captive populations, suggesting the wild population has also experienced a recent bottleneck. These *M* values are also consistent with those found in other species with a known history of demographic reduction, which ranged from 0.60–0.69 [35]. Recent declines in the wild population of *A. lila* have been largely attributed to heavy predation, but populations devastated by predation and over-collection may also be experiencing bottleneck effects. Low density and fragmentation in the remaining wild populations may decrease mating options, increase inbreeding and rates of self-fertilization, and further decrease the likelihood of recovery.

Lessons learned from the patterns of demographic and genetic change in the captive population will be used to better manage wild populations. Wild *A. lila* will soon be placed into a newly-built predator exclosure near the southern sampling location (Fig 1). This exclosure will prevent immediate extirpation due to predation, but the cessation of gene flow and limited immigration and emigration opportunities raise concerns about inbreeding depression [36] and long-term viability, particularly considering the rapid decline of the captive population. The results of our study suggest enhanced gene flow between wild snails and those kept in the exclosure will be important to maintain genetic diversity in the exclosure population [37].
